# TRPV1 acts as a Tumor Suppressor and is associated with Immune Cell Infiltration in Clear Cell Renal Cell Carcinoma: evidence from integrated analysis

**DOI:** 10.7150/jca.45918

**Published:** 2020-07-25

**Authors:** Long Zheng, Xiaojie Dou, Huijia Song, Ruixia Gao, Xiaoshuang Tang

**Affiliations:** 1 Department of Nuclear Medicine, the Second Affiliated Hospital of Xi'an Jiaotong University, Xi'an, Shaanxi 710004, China.; 2 Department of Urology, the Second Affiliated Hospital of Xi'an Jiaotong University, Xi'an, Shaanxi 710004, China.; 3 School of Science, Xi'an Jiaotong University, Xi'an, Shaanxi 710049, China.

**Keywords:** TRPV1, clear cell renal cell carcinoma, survival analysis, immune cell infiltration, bioinformatic analysis

## Abstract

Differential expression of TRPV1 has been detected in many cancer types, including clear cell renal cell carcinoma (ccRCC). However, the clinical significance of TRPV1 expression profile in ccRCC has not been comprehensively elucidated. In this study, TRPV1 expression in ccRCC and other cancer types was analyzed based on data from the GEO and Oncomine databases. Immunohistochemical (IHC) staining was performed for further validation in human ccRCC tissue chips. Survival and correlation analyses of TRPV1 were conducted using Kaplan-Meier Plotter (KM-Plotter) and the Tumor IMmune Estimation Resource (TIMER) database. TRPV1 exhibited a low expression profile in 2 GEO datasets (GSE6344, GSE36895) and 4 Oncomine datasets (Gumz, Lenburg, Beroukhim 1 and Beroukhim 2), as also confirmed by IHC staining. Survival analysis indicated that high enrichment of TRPV1 significantly predicted a better overall survival (OS) and disease-free survival (DFS) of 1, 3, 5 and 10 years in ccRCC patients. TIMER analysis showed that TRPV1 copy number alterations (CNA) were closely related to immune cell infiltration. The detailed results indicated that TRPV1 expression was positively correlated with the infiltration level of CD4+ T cells, but negatively correlated with B cells, macrophages, and dendritic cells infiltration. In addition, TRPV1 might also be inversely related to abundance of the regulatory T cells (Treg) and the M2 subset of macrophages. Finally, we found that TRPV1 expression was tightly associated with several key molecules of the classical pathways in ccRCC, such as VHL, TP53, HIF1A, MTOR, MAPK1, MET, CTNNB1, etc. Our research work suggests that TRPV1 is a novel tumor suppressor and prognosis marker for ccRCC and is of great value for further exploration.

## Introduction

With the expansion of imaging technology in recent years, the number of patients who were diagnosed renal cell carcinoma (RCC) has rapidly increased 1. In the United States, the estimated number of new cases of RCC was 65,340 in 2018 2, but rose to 73,820 after one year 3. Clear cell renal cell carcinoma (ccRCC) is the most common pathologic subtype, accounting for at least 80% percentage of RCC. Due to an innate resistance to traditional radio- and chemo-therapies, RCC has become one of the most lethal urological tumors. Although appropriate applications of surgery or targeted therapies have prolonged survival time for ccRCC patients 4, the overall treatment effect is unsatisfactory in the long view. It is widely accepted that carcinogenesis of ccRCC involves complex dysfunction processes on the genomic level 5, and thus various molecules that might be of prognostic or therapeutic value were researched in previous studies, such as TOP2A 6, PLIN3 7, and miR-566 8. To date, the comprehensive mechanisms of ccRCC remain unclear, and novel target molecules for treatment are urgently needed.

The tumor microenvironment (TME) is a local internal ecosystem composed by immune cells, stromal cells, tumor cells, and secreted cytokines and chemokines 9. The TME has been reported to regulate cancer initiation and progression and is valuable in prognostic 10. Immune cells, as important components of the TME, have been proved to be closely associated with the clinical outcome of cancer, and considered as effective targets for anti-cancer therapies 11.

Transient receptor potential vanilloid type 1 (TRPV1), first discovered on the synaptolemma of peripheral sensory nerve fibers, is a ligand-gated, selective calcium ion channel responsible for thermoception and nociception when activated by multiple stimuli including temperature (>43°C), pH (<6.8), osmotic pressure and endotoxins 12, 13. Furthermore, TRPV1 is also regulated by ex- or endogenous chemicals belonging to the vallinoid family, such as capsaicin, piperine and geraniol 14. Despite its primarily expressed location, functional expression of TRPV1 was also proved in several cancers such as breast cancer 15, endometrial cancer 16, bladder cancer 17, colorectal cancer 18, and prostate cancer 19, among others. Previous studies found that TRPV1 was also expressed in immune cells, such as macrophages, dendritic cells, and T cells and appeared to be a modulator of immune cell function 20. However, the exact role of TRPV1 in immune systems is not wholly determined. In ccRCC, TRPV1 was demonstrated a decreased expression but negatively correlated with tumor grades and subtypes in study by Wu et al. 21, and its detailed effects on the origin, progression and TME of ccRCC are currently unsolved.

In this study, with the goal of preliminary exploration of the mechanism of TRPV1, we verified its expression profile in ccRCC based on data from immunohistochemical (IHC) staining and the GEO database. Then, the associations of TRPV1 with survival and immune cell infiltration were respectively analyzed by application of Kaplan-Meier (KM) plotter and Tumor IMmune Estimation Resource (TIMER), two website servers for bioinformatic analysis. Finally, we computed the correlation of TRPV1 expression with key molecules of several classic pathways involved in the origin, progression and TME of ccRCC.

## Materials and Methods

### Tissue specimen and reagents

The two tissue chips (No. BC07014a and OS-Kid01002) of RCC totally containing 27 normal tissues and 63 ccRCC tissues were purchased from Alenabio (Xi'an Alena Biotechnology Ltd., Co.). TRPV1 primary antibody (HT-150, goat anti rabbit poly-antibody) was purchased from Santa Cruz (Santa Cruz Biotechnology, Inc.). The Ultra-sensitive^TM^S-P IHC Kit (No. KIT-9720) and DAB chromogenic kit (No.0031-1031) were sourced from Maixin Biotechnology in the city of Fuzhou, China.

### Expression analysis in Oncomine and GEO Database

Oncomine is one of the most famous cancer microarray databases, containing 715 datasets and 86,773 samples, and serves as a data-mining platform 22 (https://www.oncomine.org/resource/login.html). GEO was built by the National Center for Biotechnology Information (NCBI), and is a database of gene expression data and an online genomic resource that collects high-throughput gene expression data uploaded by research institutes around the world (https://www.ncbi.nlm.nih.gov/geo/).

Three ccRCC genesets (GSE6344, GSE36895, GSE781) were downloaded from GEO. TRPV1 expression raw data were extracted for further differential analysis between normal tissue (NT) and ccRCC tissue. In the Oncomine database analysis, TRPV1 was input as a gene symbol and diverse comparing methods were chosen according to the analysis purposes. The following filter conditions were set: *P*-value less than 0.05, fold change greater than 1.5, and gene rank of all.

### Prognostic analysis in Kaplan-Meier Plotter database

The research on the TRPV1 prognostic value was performed in the KM-plotter database, which is capable of evaluating the survival of more than 54,000 genes in 21 cancer types 23. The ccRCC dataset with 530 samples was selected to explore the expression profile of TRPV1 on ccRCC overall survival (OS) and disease-free survival (DFS). Furthermore, the hazard ratio (HR), log-rank P-value and survival plots were computed and output by the website automatically.

### TIMER database Analysis

As previously reported, immune cells in the TME were tightly related to the clinical outcome of cancer, and TRPV1 seemed to be an important modulator of immune cell function. We decided to study the interaction between immune cell infiltration and TRPV1 expression using the Tumor IMmune Estimation Resource (TIMER) database. Based on data from TCGA, TIMER is a comprehensive web server for prediction immune cell infiltration in various tumor types and supplies multiple modules of analysis on the abundance of immune infiltrates such as genes, survival, correlation and other factors 24. This study researched the TRPV1 profile in ccRCC and the correlation of TRPV1 expression with infiltration degree and recognized gene markers of common immune cells consisting of B cells, CD4^+^ T cells, CD8^+^ T cells, neutrophils, macrophages, and dendritic cells. The relationships of TRPV1 expression with the key molecules of common pathways were also evaluated using the correlation module in TIMER. The coexpression data were computed by Spearman's correlation analysis, and scatter plots with correlation index and *P*-value were constructed automatically.

### Immunohistochemical (IHC) staining analysis

The tissue chips were treated with heat, dewaxing, and hydration, followed by an antigen repairing process under microwave and Tris-EDTA buffer (pH=8.0) conditions. After blocking of endogenous peroxidase and nonspecific binding sites, anti-TRPV1 antibody was applied to the tissue chips with a titer of 1:100 and allowed to react for 24h at 4 centigrade. Following with secondary antibody reaction, the chromogenic reaction was performed with DAB reagent. Finally, a series of operations was performed to acquire the IHC stained chip, involving hematoxylin restaining, ammonium hydroxide rebluing, hydrochloric acid differentiation, and resin sealing. The prepared chip was observed under a microscope. The IHC score was defined as the product value of the stain intensity (0~3) and portion of stained cells (1~4).

### Statistical analysis

The differential TRPV1 expression level or IHC score between normal tissue and ccRCC was evaluated by the independent* t*-test. One-way ANOVA was used to analyze the difference of grades and T stages in ccRCC samples. The *P*-values <0.05 was applied to justify the statistical significance. Correlation analysis of TRPV1 expression with the infiltration level and biomarkers of immune cells was evaluated by Spearman's score and statistical significance. The intensity of correlation was defined as follows: 0.00-0.29 “weak”, 0.30-0.49 “moderate”, 0.50-0.79 “strong”, and 0.80-1.00 “very strong”.* P*-values <0.05 was considered statistically significant.

## Results

### TRPV1 expression profiles in diverse cancer types

**Figure 1:**
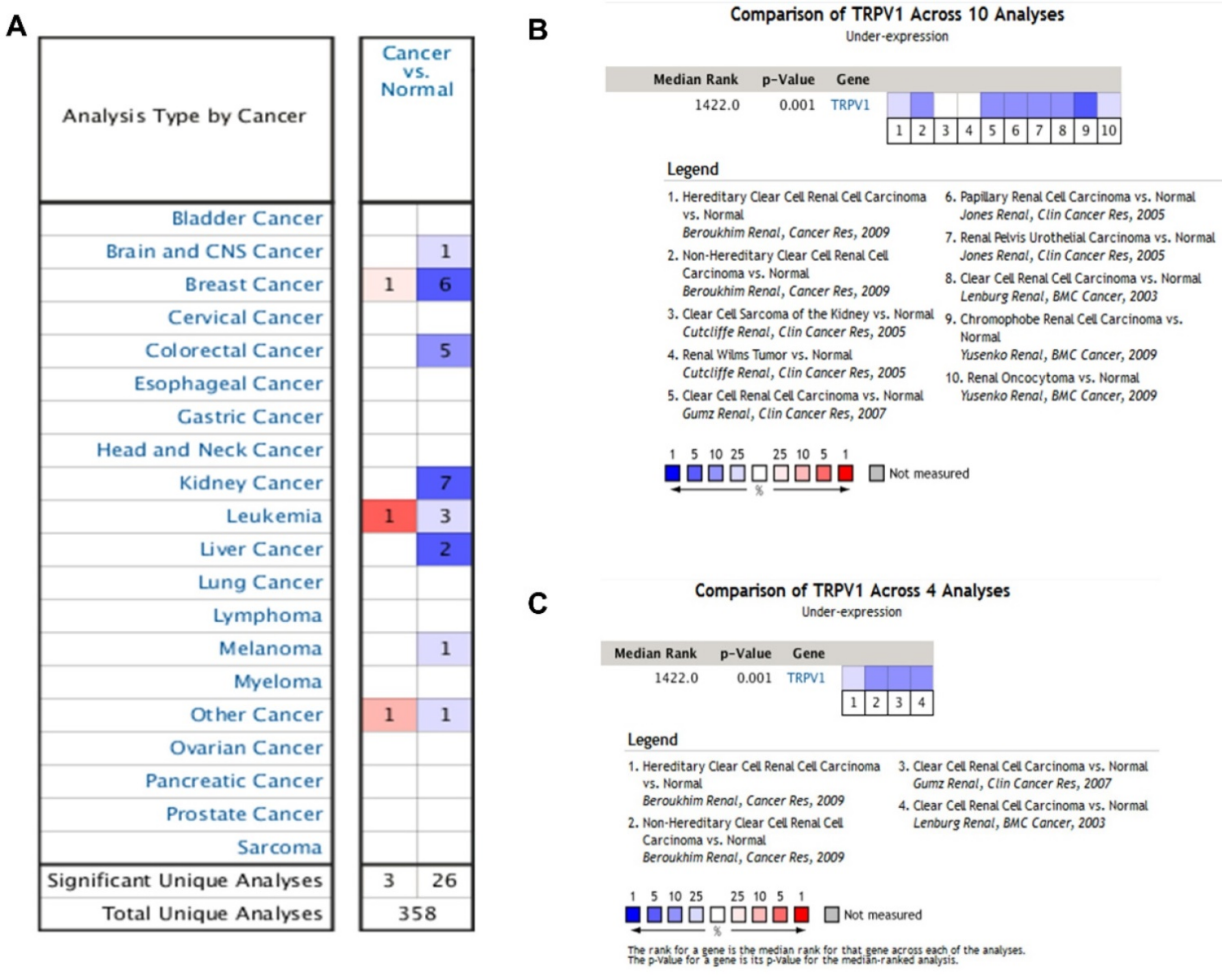
** TRPV1 expression analysis in Oncomine database.** TRPV1 expression level of different cancer types compared with normal tissues (**A**); Summary analysis of TRPV1 expression in 10 renal cell carcinoma datasets (**B**), and 4 clear cell renal cell carcinoma datasets (**C**). The color blue represents low expression, and red represents high expression.

According to the results from the Oncomine database, the profiles of TRPV1 expression varied in different cancers. TRPV1 was expressed at low levels among breast cancer, colorectal cancer, kidney cancer, liver cancer, melanoma, and brain cancer. In contrast, high expression was observed in breast cancer, leukemia, and other cancers (Figure 1A). The meta-analysis result also indicated downregulation of TRPV1 in 10 genesets of multiple kidney cancers (Figure 1B) and 4 genesets of ccRCC (Figure 1C), and the summary *P*-values were 0.001.

### TRPV1 was expressed at low levels in ccRCC by bioinformatic analysis and IHC staining

**Figure 2:**
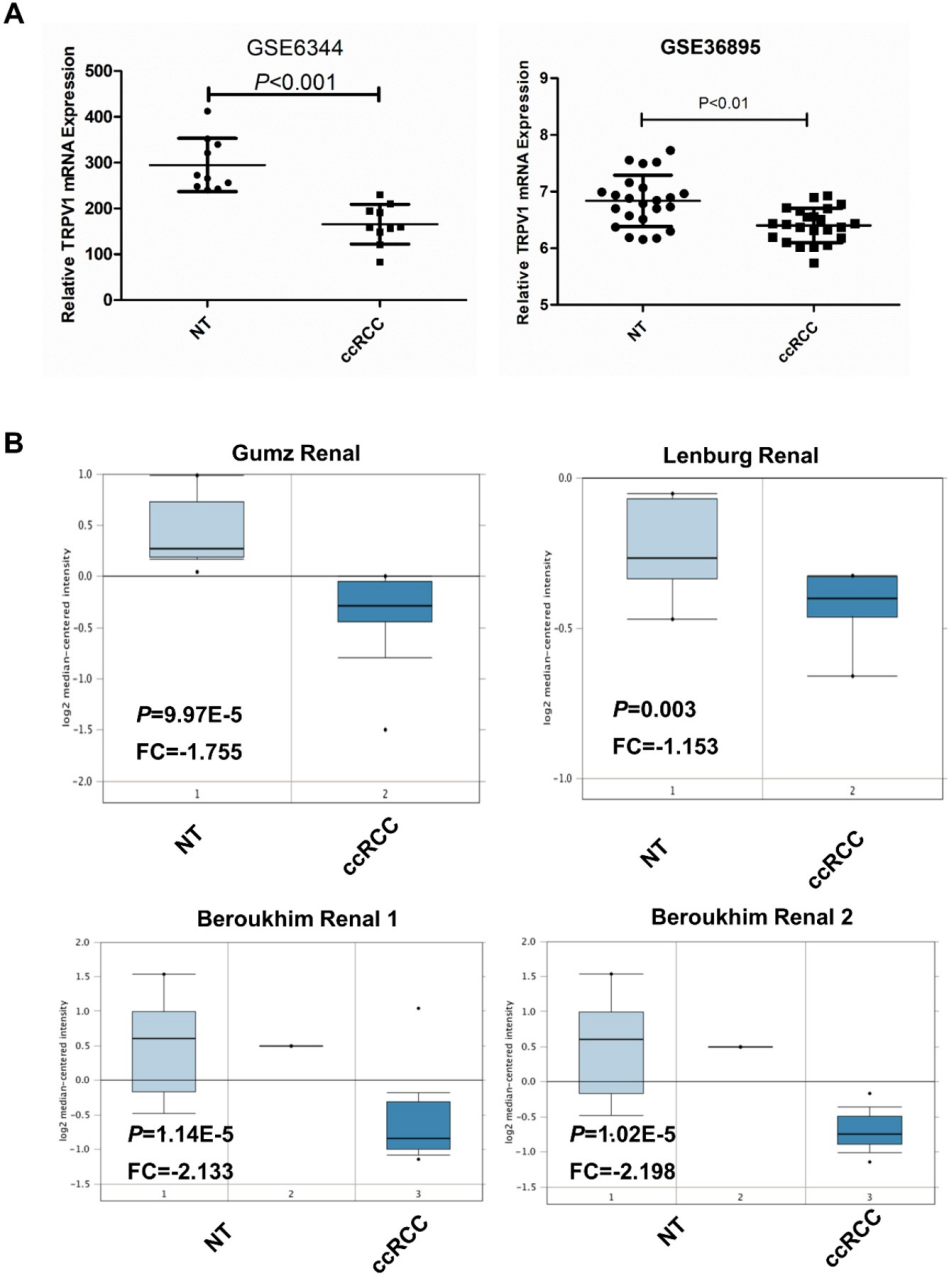
TRPV1 expression in ccRCC datasets including 2 GEO datasets (GSE6344, GSE36895) (**A**) and 4 Oncomine datasets (Gumz, Lenburg, Beroukhim 1 and Beroukhim 2) (**B**).

**Figure 3:**
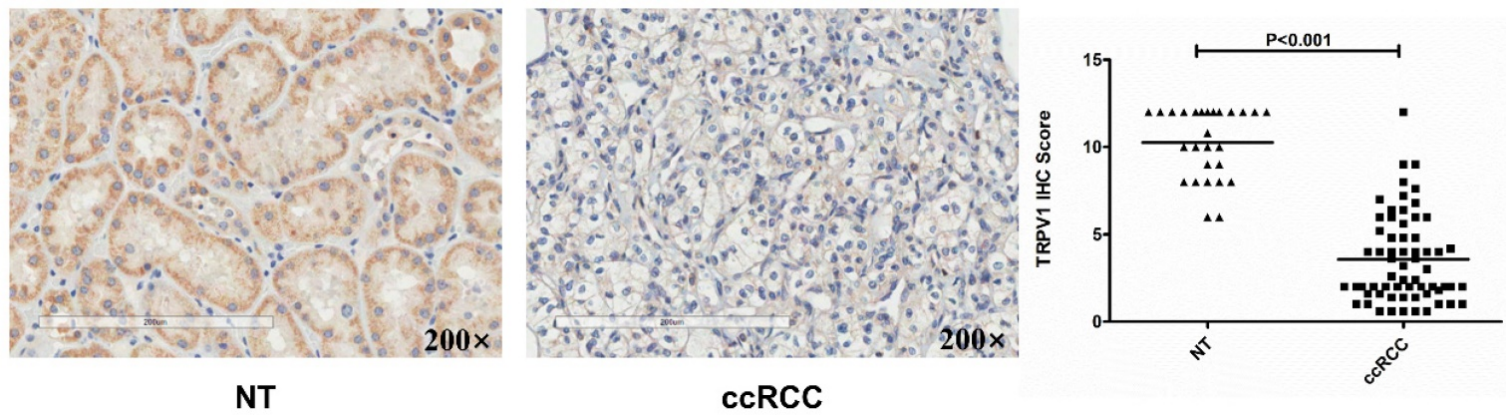
Immunohistochemical staining of TRPV1 in human ccRCC and normal tissues. NT: normal tissue, ccRCC: clear cell renal cell carcinoma.

Based on data from the two GEO (GSE6344, GSE36895) and three Oncomine geneset (Gumz, Lenburg, Beroukhim), we found that TRPV1 expression was significantly lower in ccRCC than it in NT (Figure 2). Although TRPV1 downregulation of ccRCC was also observed in GSE781, no statistical significance was found with *P*=0.091 (Figure S1). For further validation, we applied IHC staining in two tissue chips containing 27 NT and 63 ccRCC. The results demonstrated that TRPV1 positive cells exhibited strong yellow or brown staining and were enriched in the epithelial cells of renal tubes, whereas weak staining or even nonstaining was observed in ccRCC tissues. IHC staining showed a low expression profile of TRPV1 in ccRCC compared with NT (Figure 3). However, our outcome still indicated there was no relevance between TRPV1 expression and the stages or grades of ccRCC (Figure S2).

### High expression of TRPV1 predicts better OS and DFS in ccRCC

**Figure 4:**
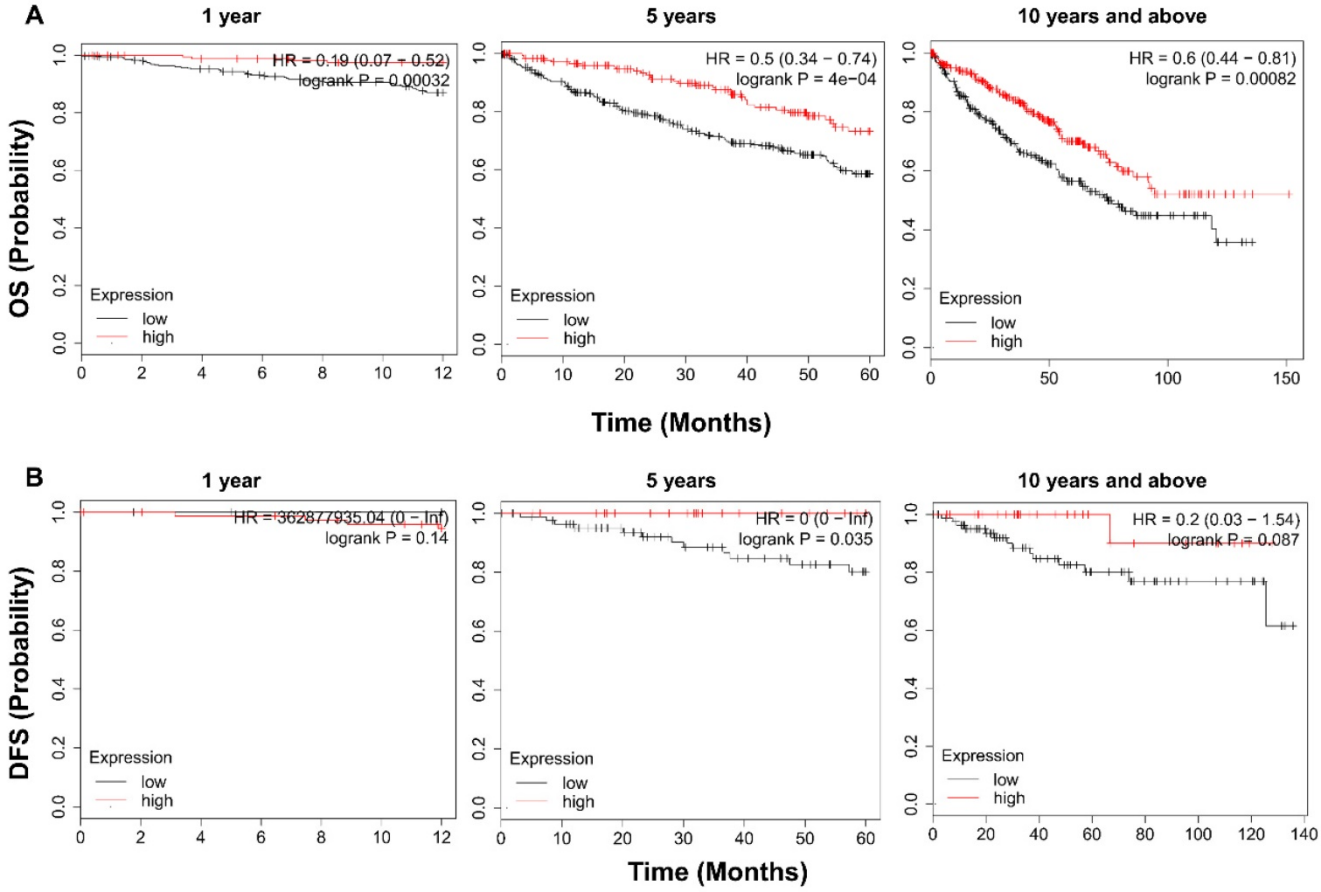
** Kaplan-Meier survival analysis of TRPV1 in ccRCC.** (**A**) Overall survival. (**B**) Disease free survival.

To examine the prognostic value of TRPV1, KM survival analysis was conducted. As shown in Figure 4A, ccRCC patients with enriched TRPV1 expression had a significantly longer time of OS in 1, 5, 10 and >10 years. For DFS, a high TRPV1 level still predicted better outcomes, but only the 5-years DFS was of statistically significant (Figure 4B).

### TRPV1 profile is associated with immune cell infiltration level

**Figure 5:**
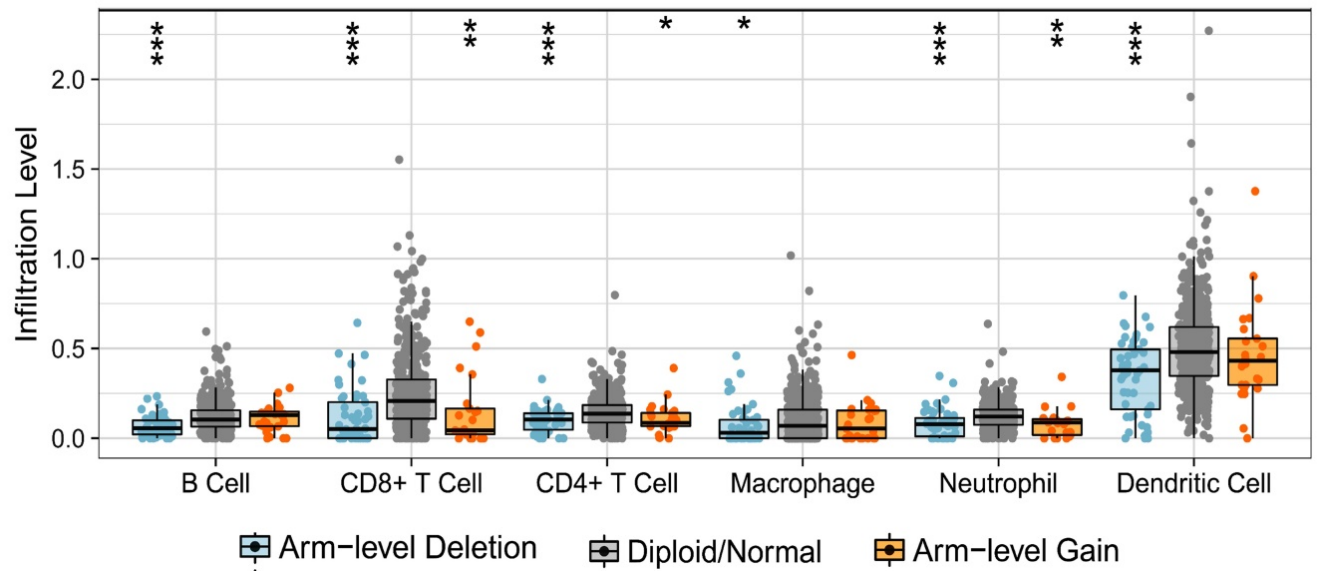
** Correlation of TRPV1 CNA and immune cell infiltration.** CNA: copy number alterations.

Firstly, we analyzed the effect of TRPV1 copy number alteration (CNA) on the infiltration level of six kinds of immune cells. A decrease in the TRPV1 gene copy number (arm-level deletion) significantly alleviated infiltration intensity among immune cells, e.g., B cells, CD4^+^ T cells, CD8^+^ T cells, neutrophils, macrophages, and dendritic cells. Furthermore, an increase of TRPV1 CNA (arm-level gain) was also observed to be negatively related to the infiltration level of CD4^+^ T cells, CD8+ T cells, neutrophils, and dendritic cells (Figure 5). These discoveries indicated that TRPV1 appeared to be involved in regulation of immune infiltration in ccRCC.

**Figure 6:**
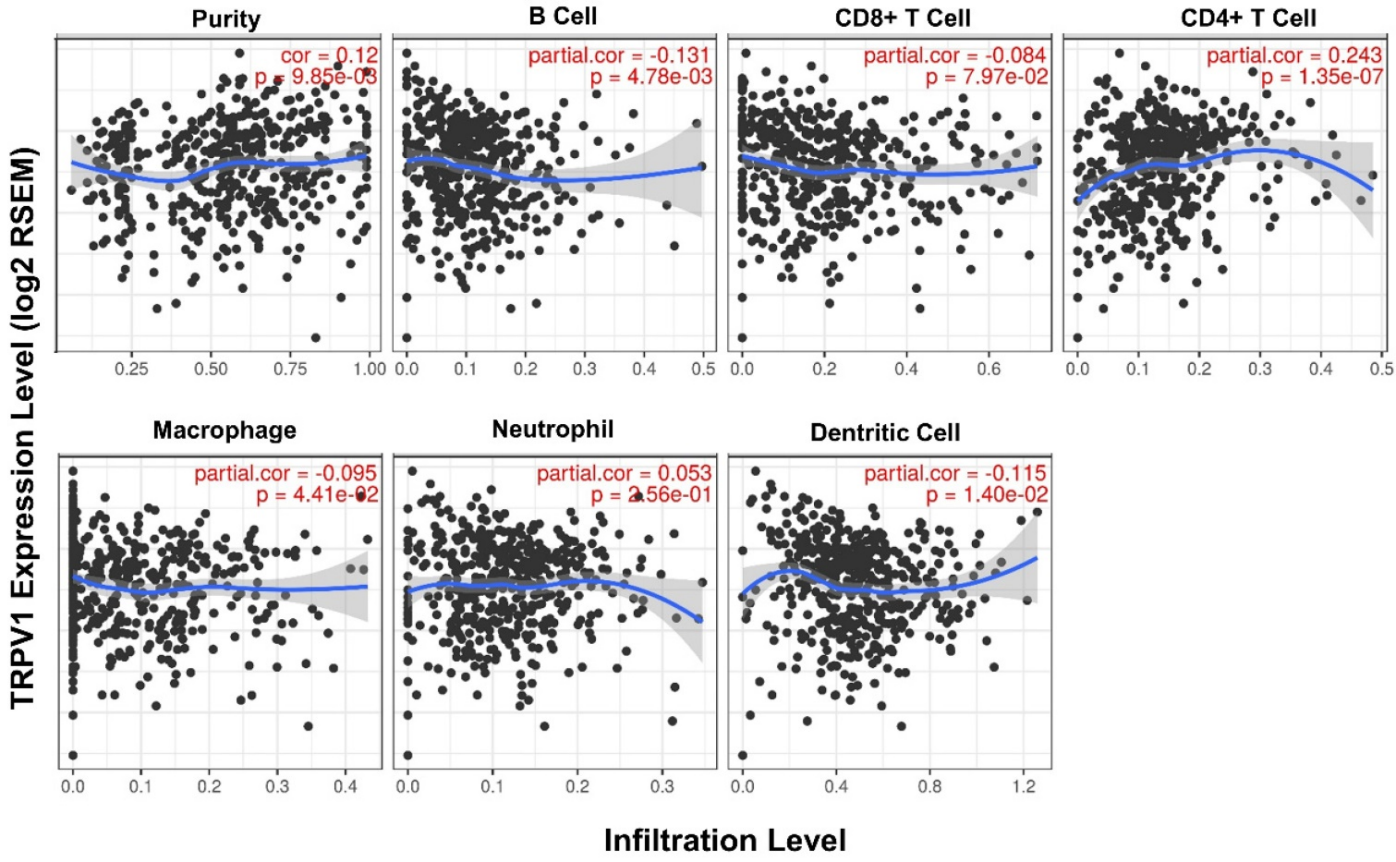
Correlation between TRPV1 expression and infiltration level of immune cells.

Second, the quantified correlation between TRPV1 expression and immune cell infiltration was further analyzed. The TRPV1 expression level had weak negative correlations with the infiltration level of B cells (*r* = -0.131, *P* = 4.78e-03), macrophages (*r* = -0.095, *P* = 4.78e-03), and dendritic cells (*r* = -0.115, *P* = 1.40e-02) and also had a weak positive correlation with CD4^+^ T cells (*r* = 0.243, *P* = 1.35e-07) (Figure 6). All correlations were statistically significance with a definition of *P*<0.05.

Third, the effects of immune cells on ccRCC survival were analyzed. As shown in Figure S3, a high infiltration level of CD8^+^ T cells, CD4^+^ T cells and macrophages predicted a longer survival time in 3 and 5 years. We also conducted another survival analysis between TRPV1 expression and cumulative survival in each tumor immune subset. The outcome showed that high TRPV1 expression was associated with better 3-year or 5-year cumulative survival time in the CD8^+^ T cell, CD4^+^ T cell and macrophage subsets of ccRCC (Figure S4).

### TRPV1 expression is correlated with the differentiation of macrophage and T cell subsets

**Figure 7:**
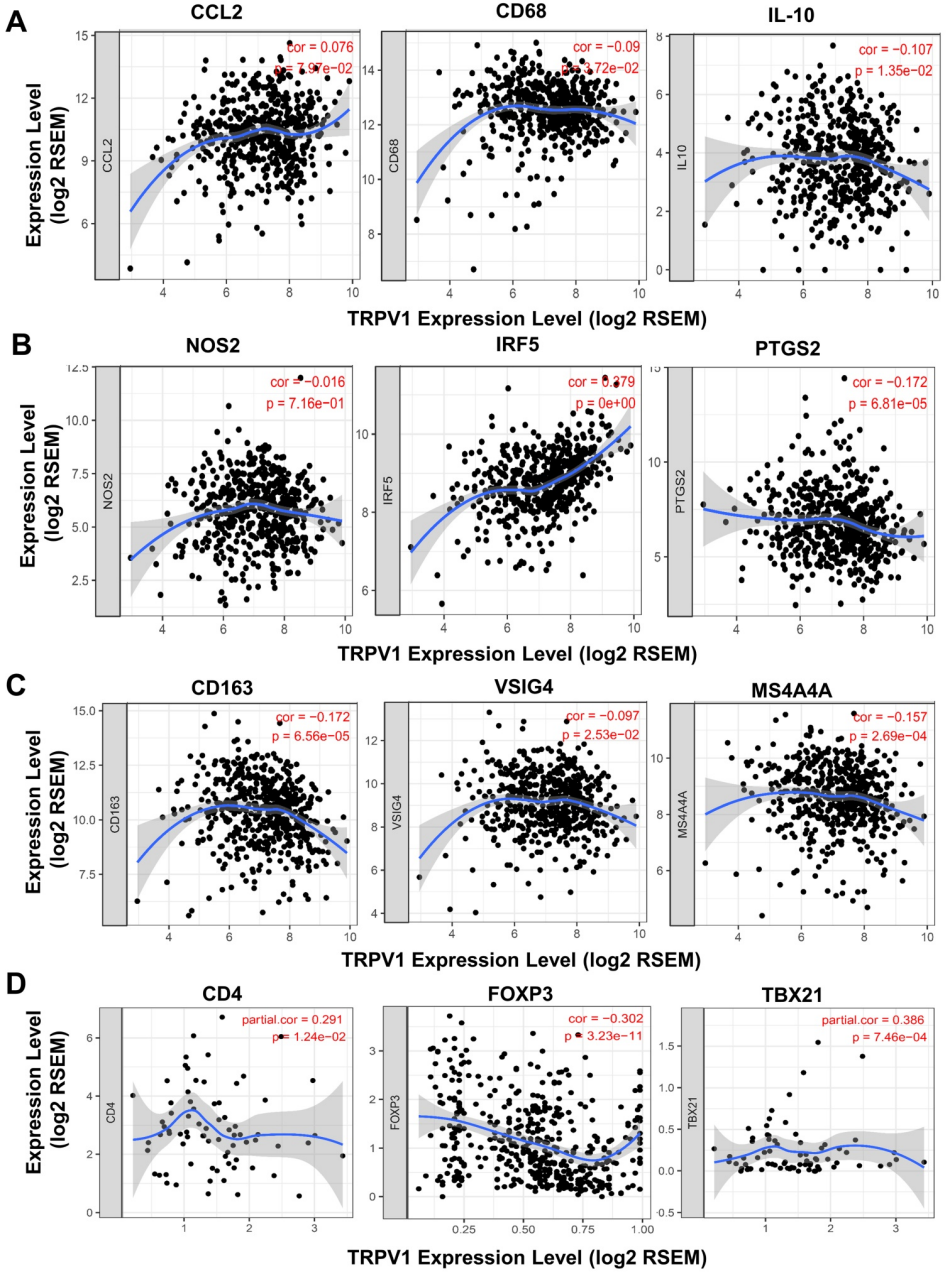
** Correlation of TRPV1 expression with biomarkers of macrophage and T Cells.** (**A**) Tumor associated macrophages (TAM), (**B**) M1 subtype, (**C**) M2 subtype, and (D) T Cells.

A recent study reported that T cells and macrophages were tightly associated with clinical outcomes of ccRCC 25. We decided to explore the correlation of TRPV1 expression and the differentiation status of macrophage and T cell subsets. Several biomarkers of macrophage and T cell subsets were introduced according to the studies of Pan 26 and Xu 27. In Figure 7A, TRPV1 was shown to be weakly and negatively correlated with expression of CD68 (*r* = -0.09, *P* = 3.72e-02) and IL10 (*r* = -0.107, *P* = 1.35e-02), which are the biomarkers of tumor associated macrophage (TAM). It is well-known that macrophages are clarified into 2 subsets of M1 and M2 based on polarization status. We found that TRPV1 expression had a weak positive correlation with M1 marker IRF5 (*r* = 0.279, *P* = 0e+00) and was negatively correlated with PTGS2 (*r* = -0.172, *P* = 6.81e-05) (Figure 7B). Additionally, our results indicated that TRPV1 expression had negative weak relationships with M2 markers CD163 (*r* = -0.172, *P* = 6.56e-05), VSIG4 (*r* = -0.097, *P* = 2.53e-02) and MS4A4A (*r* = -0.157, *P* = 2.69e-04) (Figure 7C).

The CD4^+^ T cells could be divided into two subsets, namely, regulatory T cells (Treg) and helper T cells (Th). As shown in Figure 7D, a negative moderate association (*r* = -0.302, *P* = 3.23e-11) between TRPV1 and Foxp3 (Treg cell marker) was observed, comparing with another positive moderate correlation (*r* =0.386, *P* =7.46e-04) with TBX21 (Th cell marker).

### TRPV1 expression profile is related to the activation state of different signaling pathways

**Figure 8:**
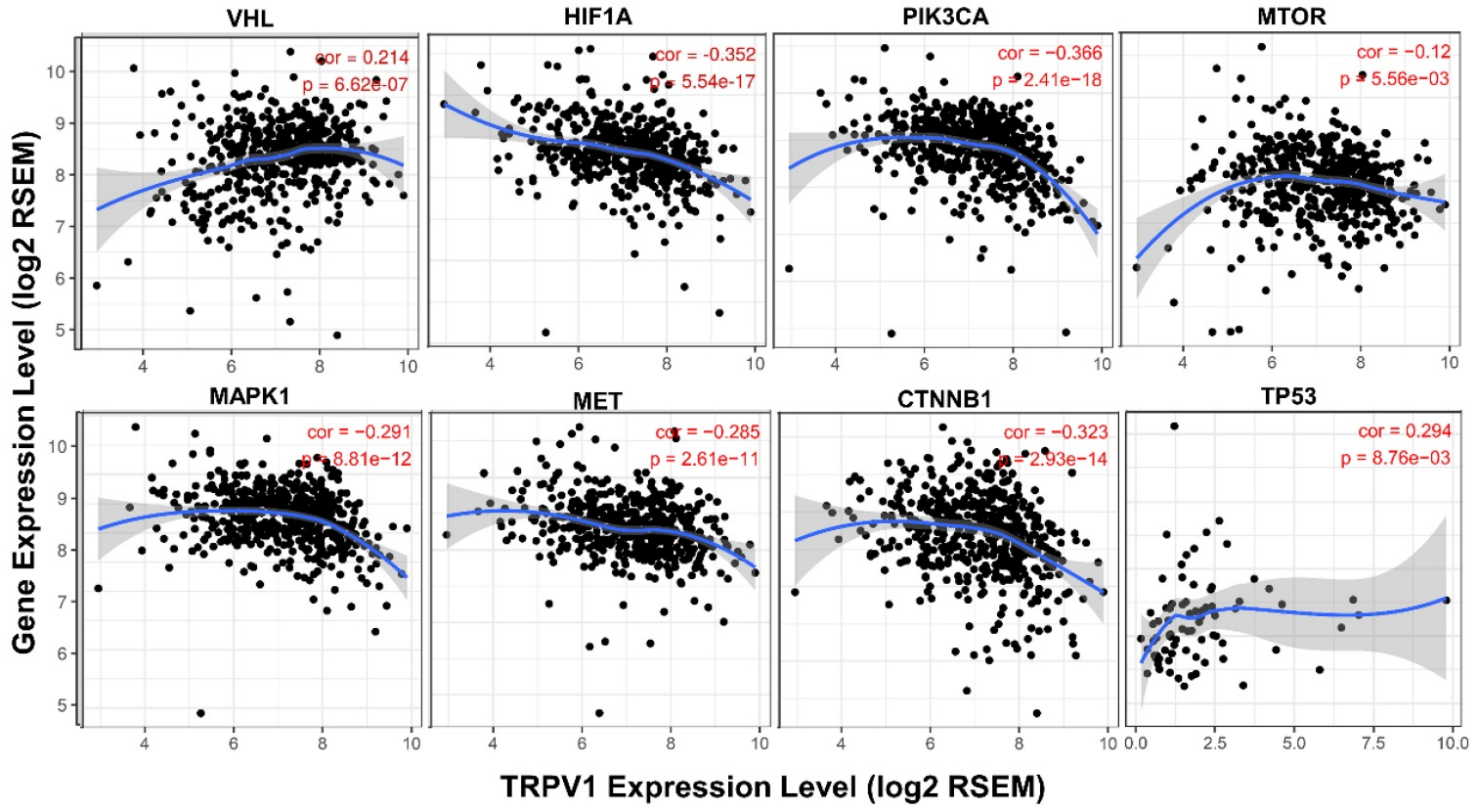
Correlation of TRPV1 expression with key molecules of different pathways.

It is well known that various pathways play crucial roles in generation and development of ccRCC, and we further analyzed the association between their activation states and TRPV1 expression. From the results shown in Figure 8, we found that the TRPV1 profile was positively correlated with expression level of VHL and TP53, known as cancer suppressor genes, and had a negative relationship with well-known cancer promoting genes such as HIF1A, PIK3CA, MTOR, MAPK1, MET, CTNNB1. However, our outcomes also showed certain discrepancies in TRPV1 expression and other cancer related genes. One factor was inconsistent correlation among TRPV1 and selected Wnt family members (WNT3A, WNT5A, WNT1, WNT2). The other factor was TRPV1 downregulation aberrantly correlated with PTEN (*r* = -0.206, *P* = 1.63e-06), a classic tumor suppressor, and VEGFA (*r* = -0.493, *P* = 0e+00), which is widely viewed as a promoting gene of ccRCC (Figure S5).

## Discussion

The results of our current study indicated that TRPV1 acted as a tumor suppressor gene in ccRCC according to discoveries from the following 4 aspects. First, TRPV1 expression was significantly lower in ccRCC than NT. Second, high expression of TRPV1 predicted better survival outcomes, whether OS or DFS. Third, TRPV1 CNA was of closely relevant to immune cell infiltration level, and further analysis indicated that TRPV1 expression was negatively correlated with infiltration levels of macrophages, B cells and dendritic cells but positively correlated with CD4^+^ T cells. Finally, the enrichment of TRPV1 was inversely related to certain tumor promoting genes and positively correlated with several cancer inhibiting genes.

Several researchers also focused on the tight relationship between TRPV1 and cancer. A similar expressive profile of TRPV1 was also demonstrated in other cancer types, such as colorectal cancer 18 and hepatocellular carcinoma 28. Furthermore, the study by Miao et al found highly expressed TRPV1 coupled with better DFS, which was consistent with our finding. However, another article launched by Lozano et al considered that the effect of TRPV1 on cancer survival was not completely dependent on expression quantity but on the distribution pattern 29. Those researchers defined two patterns: one is the “classical category” that exhibited diffuse expression in whole cells, and the other is the “nonclassical category” that is expressed in aggregates at the ER/Golgi structures. According to those results, the classical category predicted higher survival rate, and the nonclassical category represented poor survival. Due to tumor heterogeneity and complexity, the comprehensive role of TRPV1 in affecting ccRCC survival requires additional research to elucidate the details.

Our current research indicated that TRPV1 expression profile was associated with immune cell infiltration. High expression of TRPV1 predicted an elevated infiltration level of CD4^+^ T cells, which was validated by our discovery that TRPV1 was positively correlated with CD4 marker. Although the tumor promoting function of CD4^+^ T cells was reported in a study by Wang et al 30, we consider that this inverse conclusion may be due to the influence of Treg cells, which are a subpopulation of CD4^+^ T cells and have been discovered to facilitate tumor progression by inhibiting the anti-tumor effects of CD4^+^ T cells 31. As documented in a review by Chen et al, Th cells can exert both tumor-suppressive effects, as determined by their effector functions 32. According to our results, TRPV1 expression was negatively correlated with Treg marker but positively with Th markers, suggesting that TRPV1 could inhibit the abundance of Treg cells while facilitating Th cell infiltration.

Additionally, we speculated that TRPV1 might affect the activation and polarization of macrophages, especially the M2 subtype. A high infiltration number of tumor associate macrophages (TAM) have been proven to representing a more aggravating phenotype and worse clinical outcomes in ccRCC 33, 34. The two types of activated macrophages are M1 and M2. M1 are classically activated macrophages (CAM) and acts as a tumor suppressor in antitumor immune response 35. M2 macrophages were proven as “renegade” in immune cells and are closely related to poor clinical prognosis in diverse cancer types, including ccRCC due to their ability to produce angiogenic and immunosuppressive molecules 33. In our study, TRPV1 was found to be weakly and negatively associated with macrophages infiltration. Further analysis indicated that TRPV1 expression was significantly positively correlated with M1 biomarkers (IRF5, PTGS2) but negatively correlated with M2 biomarkers (CD163, VSIG4, MS4A4A). These results suggested that TRPV1 might induce a macrophage-related antitumor immune response by activating M1 subsets and inhibiting M2 subsets.

Recently, Zhang et al demonstrated that a high proportion of Treg cell and M2 macrophages represented poor clinical outcomes 25. Our results showed that TRPV1 is negatively correlated with Treg and M2 markers, indicating that TRPV1 may inhibit differentiation of Treg and M2 cells. As previously mentioned TRPV1 was found to be expressed in immune cells and appeared to modulate immune function through calcium or other signal pathways when activated by external stimuli (temperature, pH, agonists). As a well-known second messenger, the calcium ions play important roles in activation, differentiation, and secretion of various immune cells 36. Relevant studies have shown that changes in temperature or pH could enhance the differentiation process of T cells to effector cells 37, 38. These studies also indicated that TRPV1 activation and its downstream calcium signaling are involved in regulation of immune cell functions 20. Therefore, the exact mechanisms of TRPV1 and immune cell modulation require further exploration.

In addition, the present study showed that the correlations between TRPV1 and immune cell infiltration were weak to moderate, but had statistical significance. Similar results of weak correlation between other genes and immune cell infiltration were also reported in some researches 26, 27, 39. These findings are worth to be further validated by histopathological methods.

Previous studies have proved the several classical signaling mechanisms were involved in promoting ccRCC, such as VHL/HIF1α 40, PI3K/Akt/mTOR 41, HGF/MET 42, MAPK 43, and Wnt/β-Catenin 44, etc. TRPV1 expression was discovered to be negatively correlated with expression of the key proteins in those pathways and positively correlated to cancer inhibiting genes, p53 and VHL. In other words, TRPV1 might suppress ccRCC by inhibiting activation of those classical signaling mechanisms. Nevertheless, certain aberrant outcomes were also displayed in our analysis. TRPV1 had a negative relationship with the well-recognized cancer suppressor gene Pten and a positive relation with VEGFA, a known cancer promoting gene. In our future research work, the interaction between TRPV1 and key pathways will be validated.

As a potential tumor suppressor gene, the TRPV1 expression has been demonstrated to be regulated by exogenous stimuli or chemicals. In the studies by Ma et al. indicated acid stimulation (pH=5.0) elevated TRPV1 expression in esophageal epithelial cells 45. Gingerol and formaldehyde were demonstrated to increase TRPV1 level in the recent studies of Han 46 and Geng 47. Thus, TRPV1 appears to be a novel target with great therapeutic significance for ccRCC.

The limitation in our study is a lack of experimental evidence to support the bioinformatic analysis results. Further experimental work on verifying our discoveries is planned.

## Conclusion

In summary, our study indicates that low expression of TRPV1 is detected in ccRCC tissues and is associated with poor clinical outcomes. A significant weak to moderate correlation was found in TRPV1 expression profile, immune cell infiltration and signaling pathways activation. TRPV1 is suggested as a novel tumor suppressor and prognosis marker for ccRCC and might be of great value for further researching.

## Supplementary Material

Supplementary figures and tables.
