# Supplementary material for: TRPV1 acts as a Tumor Suppressor and is associated with Immune Cell Infiltration in Clear Cell Renal Cell Carcinoma: evidence from integrated analysis
Source: J Cancer. 2020 Jul 25;11(19):5678–88. doi: 10.7150/jca.45918 (PMC7477432; doi:10.7150/jca.45918)
Supplement: Supplementary file 1 — Supplementary figures and tables. [file jcav11p5678s1.pdf]

Supplementary Figures Legends:

Supplementary Figure 1 TRPV1 expression in GSE781.

Supplementary Figure 2 Subgroup analysis of TRPV1 expression in ccRCC. (A) Grouped by stages in GSE6344. Grouped by grades (B) and stages (C) in tissue chip (BC07014a).

Supplementary Figure 3. Correlation of immune cells infiltration and survival in ccRCC. (A) 1 year; (B) 3 years; (C) 5 years; (D) 10 years. The red line represents high infiltration of immune cells, while the blue represents low infiltration.

Supplementary Figure 4. Correlations between TRPV1 expression and cumulative survival in each ccRCC immune subsets. (A) 1 year; (B) 3 years; (C) 5 years; (D) 10 years. The red line represents high expression of TRPV1, while the blue represents low expression.

Supplementary Figure 5. Correlation of TRPV1 expression with other key molecules of different pathways.

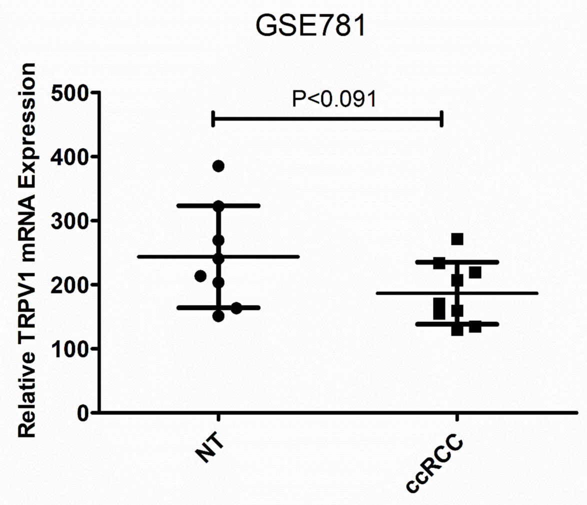

GSE781
P<0.091
Relative TRPV1 mRNA Expression
500
400
300
200
100
0
NT
ccRCC

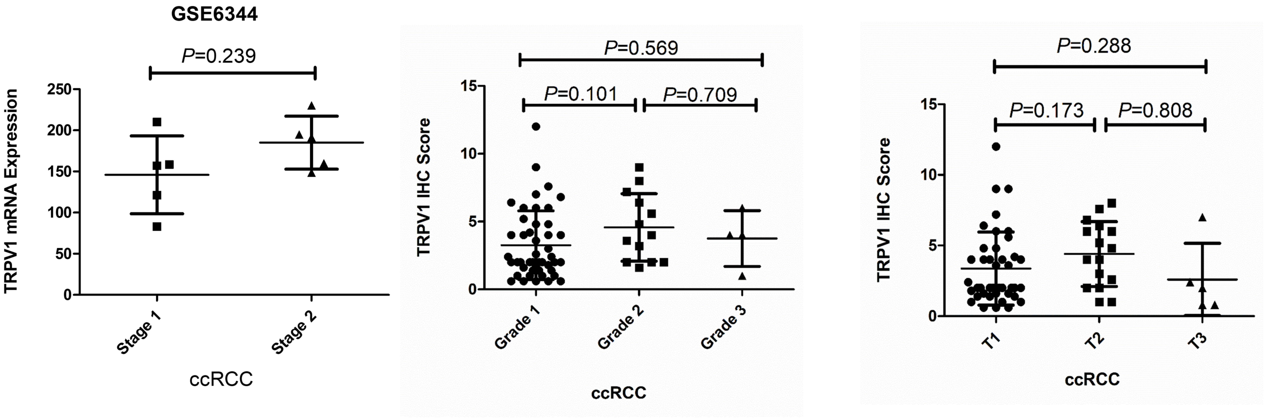

GSE6344
P=0.239
TRPV1 mRNA Expression
250
200
150
100
50
0
Stage 1
Stage 2
ccRCC
P=0.569
P=0.101
P=0.709
TRPV1 IHC Score
15
10
5
0
Grade 1
Grade 2
Grade 3
ccRCC
P=0.288
P=0.173
P=0.808
TRPV1 IHC Score
15
10
5
0
T1
T2
T3
ccRCC

A

B

C

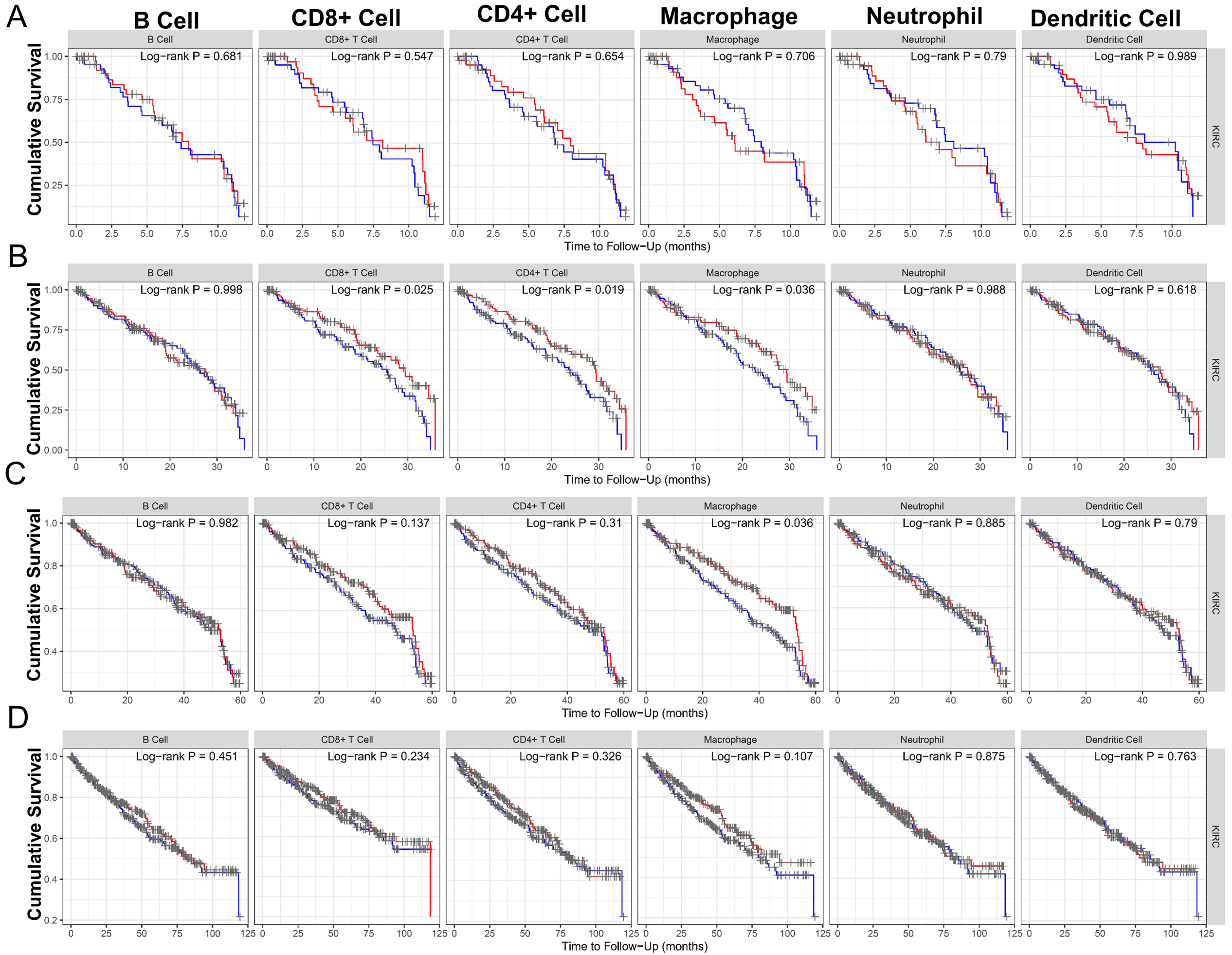
A
B Cell
CD8+ Cell
CD4+ Cell
Macrophage
Neutrophil
Dendritic Cell
CD8+ T Cell
CD4+ T Cell
Log-rank P = 0.681
Log-rank P = 0.547
Log-rank P = 0.654
Log-rank P = 0.706
Log-rank P = 0.79
Log-rank P = 0.989
Cumulative Survival
Time to Follow-Up (months)
KIRC
B
Log-rank P = 0.998
Log-rank P = 0.025
Log-rank P = 0.019
Log-rank P = 0.036
Log-rank P = 0.988
Log-rank P = 0.618
C
Log-rank P = 0.982
Log-rank P = 0.137
Log-rank P = 0.31
Log-rank P = 0.036
Log-rank P = 0.885
Log-rank P = 0.79
D
Log-rank P = 0.451
Log-rank P = 0.234
Log-rank P = 0.326
Log-rank P = 0.107
Log-rank P = 0.875
Log-rank P = 0.763

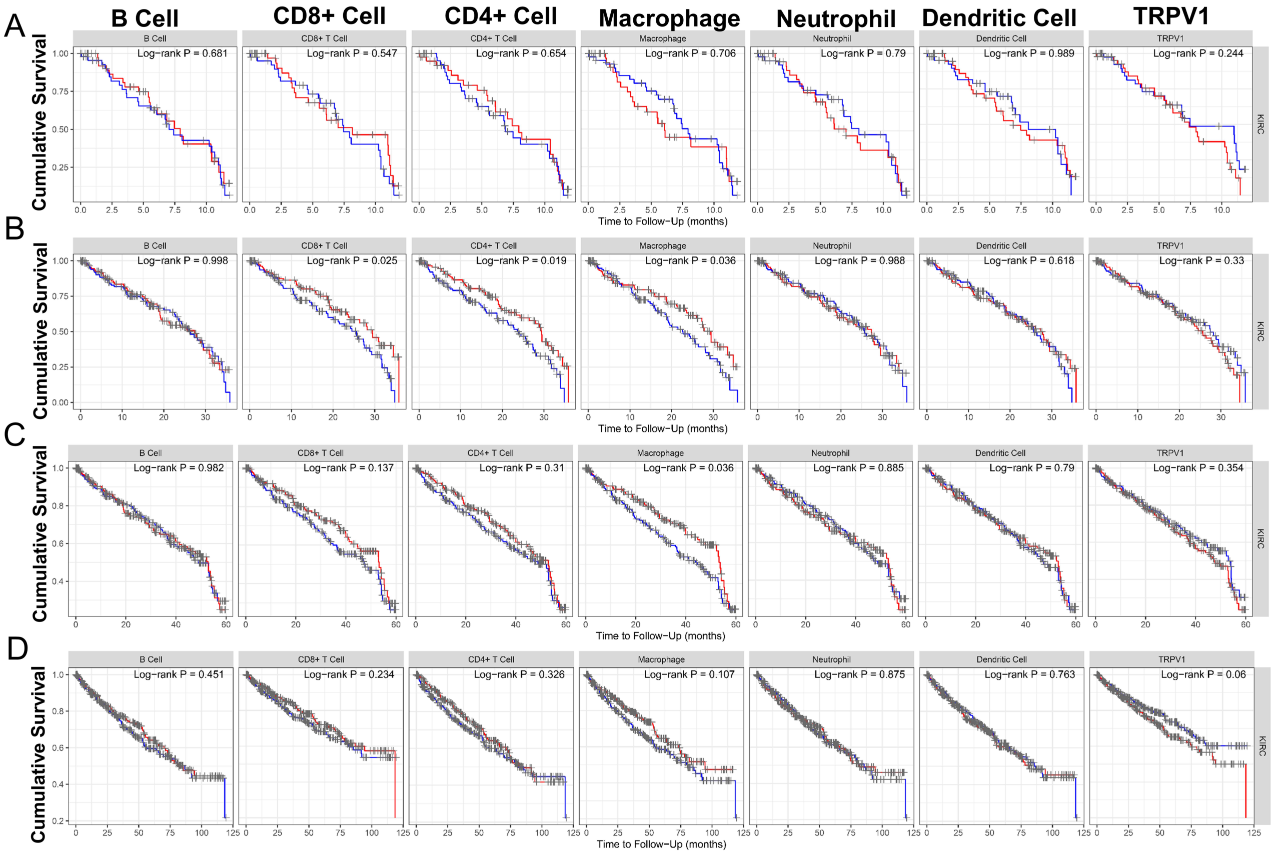
A
B Cell
CD8+ Cell
CD4+ Cell
Macrophage
Neutrophil
Dendritic Cell
TRPV1
Cumulative Survival
B Cell Log−rank P = 0.681
CD8+ T Cell Log−rank P = 0.547
CD4+ T Cell Log−rank P = 0.654
Macrophage Log−rank P = 0.706
Neutrophil Log−rank P = 0.79
Dendritic Cell Log−rank P = 0.989
TRPV1 Log−rank P = 0.244
KIRC
Time to Follow−Up (months)
B
Cumulative Survival
B Cell Log−rank P = 0.998
CD8+ T Cell Log−rank P = 0.025
CD4+ T Cell Log−rank P = 0.019
Macrophage Log−rank P = 0.036
Neutrophil Log−rank P = 0.988
Dendritic Cell Log−rank P = 0.618
TRPV1 Log−rank P = 0.33
KIRC
Time to Follow−Up (months)
C
Cumulative Survival
B Cell Log−rank P = 0.982
CD8+ T Cell Log−rank P = 0.137
CD4+ T Cell Log−rank P = 0.31
Macrophage Log−rank P = 0.036
Neutrophil Log−rank P = 0.885
Dendritic Cell Log−rank P = 0.79
TRPV1 Log−rank P = 0.354
KIRC
Time to Follow−Up (months)
D
Cumulative Survival
B Cell Log−rank P = 0.451
CD8+ T Cell Log−rank P = 0.234
CD4+ T Cell Log−rank P = 0.326
Macrophage Log−rank P = 0.107
Neutrophil Log−rank P = 0.875
Dendritic Cell Log−rank P = 0.763
TRPV1 Log−rank P = 0.06
KIRC
Time to Follow−Up (months)

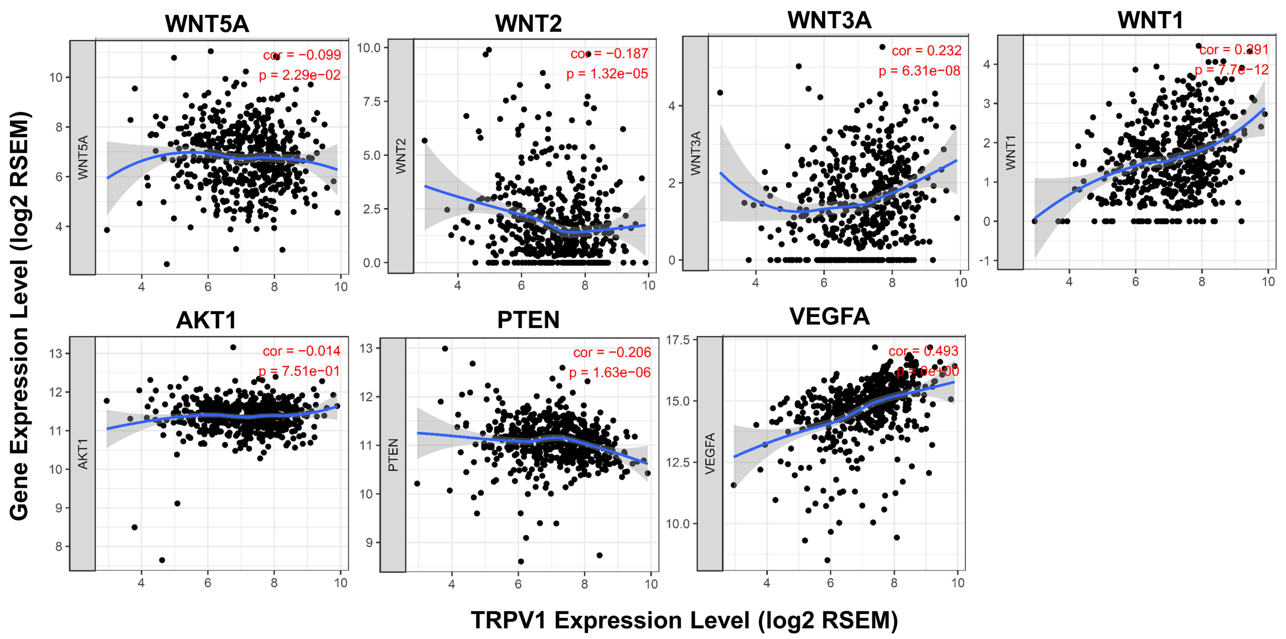
WNT5A
cor = −0.099
p = 2.29e−02
WNT2
cor = −0.187
p = 1.32e−05
WNT3A
cor = 0.232
p = 6.31e−08
WNT1
cor = 0.291
p = 7.7e−12
AKT1
cor = −0.014
p = 7.51e−01
PTEN
cor = −0.206
p = 1.63e−06
VEGFA
cor = 0.493
p = 0e+00
Gene Expression Level (log2 RSEM)
TRPV1 Expression Level (log2 RSEM)
